# Real-World Outcomes of Lip Augmentation Using a Hyaluronic Acid-Based Filler With Low 1,4-Butanediol Diglycidyl Ether Content: A Prospective, Open-Label, Multicenter, Post-marketing Study

**DOI:** 10.7759/cureus.53513

**Published:** 2024-02-03

**Authors:** Enrico Massidda, Sonia Ciampa, Ivano Iozzo, Enzo Emanuele, Piercarlo Minoretti

**Affiliations:** 1 Dermatology, Italian Society of Aesthetic Medicine, Cagliari, ITA; 2 Dermatology, Poliambulatorio Rimedical, Santarcangelo di Romagna, ITA; 3 Dermatology, Iozzo Antonucci Medical Center, Bologna, ITA; 4 Clinical Pathology, 2E Science, Robbio, ITA; 5 Occupational Health, Studio Minoretti, Oggiono, ITA

**Keywords:** butanediol diglycidyl ether, real-world aesthetic practice, lip augmentation, dermal fillers, hyaluronic acid

## Abstract

Background

1,4-butanediol diglycidyl ether (BDDE) is the most common cross-linker used to produce hyaluronic acid (HA)-based dermal fillers. However, BDDE may have cytotoxic and potentially mutagenic effects, raising safety concerns. Consequently, manufacturers are developing new HA filler formulations with reduced BDDE levels to mitigate potential biological risks. Here, we sought to evaluate the clinical outcomes of lip augmentation performed using an HA-based filler with a reduced BDDE content (Agex Fill Volume^®^; Biodue SpA, Barberino Tavarnelle, Italy) in a real-world clinical setting.

Methods

This was a prospective, open-label, multicenter, post-marketing study conducted over six months. Thirty adult subjects (29 women and one man) who desired a ≥1-point improvement on the validated Lip Fullness Scale 2 (LFS2) were enrolled. The primary efficacy endpoint was the post-procedural increase in the investigator-reported LFS2 compared to baseline. Other endpoints included self-perceived happiness assessed using the Happiness Measure Scale (HMS) and safety.

Results

Of the study participants, 73% (22/30) demonstrated an improvement of at least one point in their LFS2 scores immediately after treatment compared to baseline, thus qualifying as responders. Six months later, the responder rate, based on LFS2 scores, remained steady at 66.7% (20/30). Importantly, these aesthetic improvements were consistently associated with a positive impact on subject-reported HMS, with a significant difference (p < 0.001) between post-treatment and baseline scores. All adverse events (AEs) reported after treatment were mild.

Conclusions

Agex Fill Volume^®^, a HA filler with low BDDE content, provides a safe and effective option for enhancing lip volume in real-world aesthetic settings.

## Introduction

The labial region is not only instrumental in key functional activities such as speaking and eating, but it also significantly contributes to facial symmetry, aesthetics, and overall attractiveness [1]. Unfortunately, the lips are vulnerable to morphological changes caused by a range of factors. Intrinsic aging processes, including the natural loss of soft tissue volume, combined with external factors like exposure to ultraviolet radiation and tobacco use can significantly impact the appearance of the lips [2,3]. The orbicularis oris muscle, with its perpetual and intrinsic activity, is also responsible for the formation of perioral rhytides [4]. Gravitational forces, coupled with decreased labial volume and structural support, can cause the upper lip to elongate and migrate inferiorly [4-6]. Moreover, the decline in collagen synthesis associated with aging can diminish the definition of the vermilion border and Cupid's bow [7]. These age-related changes in labial fullness can significantly impact an individual's emotional well-being, social interactions, and self-perceived happiness [3,4,8].

Hyaluronic acid (HA)-based injectable fillers have become a leading non-surgical solution for perioral rejuvenation and lip augmentation [9]. These products have demonstrated efficacy in replenishing volume, refining morphology, and enhancing definition in the labial and perioral regions [10]. HA-based fillers are produced by synthetically cross-linking HA with specific chemicals in order to enhance their biomechanical properties and increase their retention within the body [11]. The most widely used chemical cross-linker in HA-based fillers is 1,4-butanediol diglycidyl ether (BDDE) [12]. However, several studies have indicated that BDDE can have cytotoxic and potentially mutagenic effects, raising concerns about its safety [13-16]. In response, manufacturers have developed novel HA-based filler formulations with the objective of reducing BDDE content and mitigating potential biological risks. A previous in vitro study reported that the presence of a low BDDE content in HA-based fillers can lead to an increased expression of type III collagen and elastin by cultured human fibroblasts [13]. However, this may come at the cost of reduced production of type I collagen, which is responsible for providing connective tissue firmness [13].

The commercially available filler Agex Fill Volume® (Biodue SpA, Barberino Tavarnelle, Italy) is characterized by its low BDDE content, below 0.01 ppm, and its combination of cross-linked HA with a molecular weight of two million daltons and linear HA with a molecular weight of one million daltons. These physicochemical properties make it suitable for applications beyond traditional volume restoration, such as acting as a biological modulator of the extracellular matrix (ECM) [13]. While Agex Fill Volume® is indicated for facial contouring, its safety and efficacy for perioral rejuvenation and lip augmentation have not been thoroughly evaluated in clinical practice. To address this gap, a real-world multicenter study was conducted to assess the clinical outcomes of lip augmentation using this filler. We also sought to assess patient-reported outcomes (PROs), a metric gaining recognition for its role in capturing subjective experiences related to aesthetic procedures [8,17]. Specifically, we focused on the influence of the procedure on self-perceived happiness, a significant outcome of interest to patients [18].

## Materials and methods

Study design and participants

This prospective, open-label, multicenter, post-marketing study aimed to investigate the real-world efficacy of lip augmentation with Agex Fill Volume® over a six-month period. The filler is commercially available in a pre-filled syringe containing 1.0 mL of solution with a total HA concentration of 25 mg/mL. The study enrolled adult participants (aged 18 years and older) who expressed a desire for a minimum improvement of one point on the validated Lip Fullness Scale 2 (LFS2) [19]. Exclusion criteria included pregnancy or breastfeeding, previous facial plastic surgery, facial implants, or dermal filler treatment within the past 24 months, oral surgery within the past six weeks, mesotherapy, cosmetic resurfacing, and botulinum toxin injections in the lower face below the orbital rim within the past six months. Subjects who had been on anticoagulation therapy or medication in the 10 days leading up to the procedure were also excluded. The study was carried out at three Italian aesthetic medicine centers, approved by the local ethics committee (Studio Minoretti, reference number: 2022/04HAF), and conducted in accordance with the Declaration of Helsinki. All participants provided written informed consent prior to enrollment.

Procedures

Agex Fill Volume® (1 mL) was injected into the lips using 25-gauge and 27-gauge cannulas to enhance the definition of the mucosal cutaneous border (white roll) and to augment the volume of the vermilion. In contrast, 27-gauge and 30-gauge needles were employed for the refinement of the nasal columella filters and the amelioration of perioral wrinkles, as well as to achieve lip verticalization. The application of topical anesthesia was tailored to individual patient and physician preferences.

Assessments and endpoints

Assessments were performed by the treating investigators at three-time points, i.e., before the procedure (baseline pretreatment evaluation), immediately after the Agex Fill Volume® injection, and at a six-month follow-up. Both efficacy and safety endpoints were considered. The primary efficacy endpoint was the post-procedural increase in the investigator-reported LFS2 (0 = minimal, 1 = mild, 2 = moderate, 3 = marked, and 4 = very marked) compared with baseline [19]. In addition, patients who showed an improvement of at least one point on the LFS2 compared with baseline were considered as responders [8]. Secondary efficacy endpoints included 1) investigator- and subject-reported Global Aesthetic Improvement Scale (GAIS), rated on a 5-point scale (0 = much worse; 1 = worse; 2 = no change; 3 = improved; 4 = much improved); 2) subject-reported assessment of natural look and feel of lips, rated on a 5-point Likert Scale (0 = not at all to 4 = very much); 3) subject-reported Happiness Measure Scale (HMS), an instrument used to rate subjective well-being on a 11-point Likert Scale (0 = not happy at all to 10 = extremely happy) [8,18,20]. Safety endpoints included 1) subject-reported burning, rated on a 5-point Likert Scale (0 = none to 4 = severe), 2) subject-reported pain, rated on a 5-point Likert Scale (0 = none to 4 = severe), 3) investigator-reported edema, rated on a 5-point Likert Scale (0 = none to 4 = severe), and investigator-reported erythema rated on a 5-point Likert Scale (0 = none to 4 = severe). From the time written informed consent was obtained until the conclusion of the study, a combination of bi-monthly in-person appointments and monthly phone communications was established to systematically monitor and document any adverse events (AEs).

Data analysis

To assess the normality of continuous data, we used the Kolmogorov-Smirnov test. Normally distributed variables were presented as means ± standard deviations, whereas skewed variables were expressed by medians and interquartile ranges. The paired Student’s t-test was applied for comparing normally distributed continuous variables at two different time points. When comparing three points, we used repeated measures analysis of variance (ANOVA). For skewed data, we utilized the Wilcoxon signed-rank test and the Friedman one-way repeated measure ANOVA for comparing two- and three-time points, respectively. Categorical data were presented as counts and percentages. Statistical analyses were performed using SPSS, version 20.0 (IBM, Armonk, NY, USA), with all tests two-sided at a 5% level of significance.

## Results

The study population included 30 subjects (29 women and one man) with a mean age of 43.3 ± 12.4 years. All participants successfully completed the six-month study and were, therefore, evaluable in the final analysis.

Efficacy endpoints

The efficacy endpoints observed for lip augmentation with Agex Fill Volume® are summarized in Table 1.

**Table 1:** Efficacy endpoints Data are presented as means ± standard deviations. *p < 0.001 versus baseline; ^§^p = ns versus immediately after treatment LFS2, Lip Fullness Scale 2; GAIS, Global Aesthetic Improvement Scale; HMS, Happiness Measure Scale; N/A, not available

Parameter	Baseline	Immediately after treatment	Six months
Investigator-reported LFS2	1.9 ± 0.9	2.5 ± 0.9*	2.4 ± 1.0*^,§^
Investigator-reported GAIS	N/A	2.9 ± 0.9	2.9 ± 0.7^§^
Subject-reported GAIS	N/A	3.0 ± 0.9	3.0 ± 0.8^§^
Subject-reported assessment of the natural look and feel of lips	N/A	3.8 ± 0.6	3.7 ± 0.9^§^
Subject-reported HMS	7.4 ± 1.8	9.3 ± 0.6*	9.1 ± 0.6*^,§^

**Figure 1:**
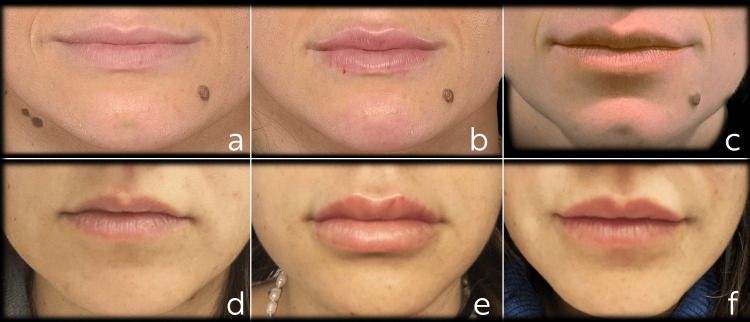
Lip appearance in two representative subjects (a,d) Pretreatment aspect, (b,e) appearance immediately after the injection of Agex Fill Volume®, and (c,f) results at the six-month follow-up visit.

On analyzing the primary outcome measure, we observed a significant increase in mean investigator-reported LFS2 scores immediately after treatment compared to baseline (p < 0.001). Furthermore, the mean LFS2 scores remained stable at the six-month mark (p = ns versus immediately after treatment; p < 0.001 versus baseline), indicating the durability of the aesthetic improvements achieved. Twenty-two subjects (73%) achieved a ≥1-point improvement in LFS2 immediately after treatment compared to baseline and were considered as responders. At six months, the LFS2 responder rate compared to baseline remained stable at 66.7% (20/30). Figure 1 shows the results obtained from two representative subjects.

**Figure 2:**
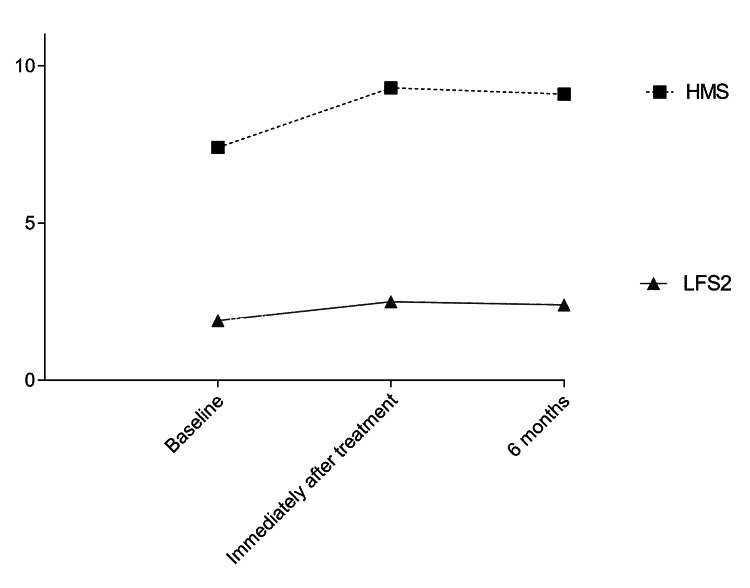
Temporal course of investigator-reported LFS2 scores and patient-reported HMS over the six-month study period A consistent alignment of subjects' self-perceived happiness with the efficacy data was observed. LFS2, Lip Fullness Scale 2; HMS, Happiness Measure Scale

The sustainability at six months of the enhancements observed immediately after treatment was corroborated by both investigator- and subject-reported GAIS scores, as well as subject-reported assessment of the natural look and feel of lips (all p = ns for six-month versus immediately after treatment comparisons). Notably, the aesthetic enhancements were accompanied by a consistently positive impact in terms of subject-reported HMS (p < 0.001 for immediately after treatment versus baseline, and p = ns for six-month versus immediately after treatment; Figure 2), indicating that lip augmentation with Agex Fill Volume® was paralleled by a substantial improvement in self-perception of happiness.

Safety endpoints

Table 2 presents the safety endpoints, indicating that no participants experienced serious AEs related to the procedure. The AEs observed immediately after treatment, including subject-reported burning and pain as well as edema and erythema assessed by investigators, were all mild in severity. Furthermore, the observed AEs showed a significant reduction at the six-month mark (all p < 0.001).

**Table 2:** Safety endpoints Data are presented as medians (interquartile range). *p < 0.001 versus immediately after treatment

Adverse event	Immediately after treatment	six months
Subject-reported burning	1 (1−1)	0 (0−1)*
Subject-reported pain	1 (1−1)	0 (0−2)*
Investigator-reported edema	1 (1−1)	0 (0−1)*
Investigator-reported erythema	0 (0−1)	0 (0−0)*

## Discussion

Prior studies have demonstrated that HA-based dermal fillers are clinically effective for lip augmentation [1,7-10]. Recent technological advances have led to the development of low BDDE crosslinked HA fillers, which are expected to have an improved safety profile [13]. However, no previous investigations have assessed the clinical outcomes of lip augmentation using HA fillers crosslinked with minimal amounts of BDDE. We, therefore, designed the current prospective, open-label, multicenter, post-marketing study of the use of Agex Fill Volume®, an HA-based filler with low BDDE content, in the lips. This article documents three principal findings. First, in terms of efficacy, we observed a significant increase in mean investigator-reported LFS2 scores immediately after treatment, which remained stable at the six-month mark, indicating durable aesthetic improvements. Notably, 73% of participants achieved the target treatment response, defined as at least a 1-point increase in the LFS2 score. A high responder rate (66.7%) was sustained at the six-month follow-up visit. Interestingly, a previous study evaluating the effectiveness and safety of a traditional HA-based filler (VYC-17.5L) for lip augmentation found that 93.2% of participants were responders at one month; however, this percentage declined significantly to 39% at 12 months [8]. This suggests that the clinical efficacy of HA fillers crosslinked with reduced amounts of BDDE, as observed in our study, is comparable to commonly used HA fillers in terms of LPS2 increase. Other investigator- and subject-reported outcomes, such as the GAIS and assessment of the natural look and feel of the lips, exhibited a similar positive trajectory throughout the six-month study period. These clinical effects were also in line with those previously observed with traditional HA-based injectable gels [8]. Second, in terms of PROs, lip augmentation with Agex Fill Volume® led to significant and long-lasting increases in patient happiness. Mean scores on the HMS were higher at both the post-treatment and six-month follow-up visits compared to baseline. This suggests that participants experienced enhanced subjective well-being following the aesthetic procedure, possibly attributable to multifaceted improvements in attractiveness, social interactions, and dating success [21]. A previous study investigated the effect of the small-particle HA filler Restylane® Silk (Galderma, Uppsala, Sweden) on self-perceived happiness after full perioral rejuvenation in 17 women [18]. Perioral treatment with Restylane® Silk significantly improved HMS scores from 7.41 ± 0.795 (pretreatment) to 7.941 ± 0.659 (post-treatment) [18]. Here, we found that Agex Fill Volume® outperformed these results. While pretreatment HMS scores (7.4 ± 1.8) were in line with those of the Restylane® Silk study, Agex Fill Volume® treatment led to markedly higher HMS scores of 9.3 ± 0.6 (after treatment) and 9.1 ± 0.6 (six-month follow-up) [18]. Notably, the alignment between the efficacy (LFS2 scores) and PROs (HMS) observed throughout the study suggests that Agex Fill Volume® injections for lip augmentation may elicit aesthetic outcomes significant enough to induce a positive and enduring emotional transformation. These findings hold particular importance considering that the HMS, while a validated measure of happiness, might not be the most optimal tool for capturing transient happiness elevation following a cosmetic procedure [18]. Finally, Agex Fill Volume® injections were well-tolerated, with participants reporting only mild post-treatment AEs that significantly diminished by the six-month follow-up visit.

The promising efficacy and safety outcomes observed in this study are likely due to the specific formulation properties of the product, specifically its low BDDE content. Prior in vitro experiments have demonstrated that HA-based fillers with reduced BDDE concentrations promote the production of type III collagen and elastin over type I collagen in human fibroblasts [13]. While type I collagen contributes to skin strength, type III collagen is associated with elasticity and suppleness [13]. Agex Fill Volume®, by inducing the synthesis of type III collagen and elastin, has the potential to function as a peculiar ECM modulator, providing a more natural and supple appearance for lip augmentation. Moreover, fillers with low BDDE content have been associated with lower levels of oxidative DNA damage in human fibroblasts, as indicated by 8-hydroxydeoxyguanosine measurements, compared to traditional HA-based fillers [13]. While this suggests that Agex Fill Volume® may offer long-term safety in lip enhancement procedures, further in vivo investigations and long-term prospective studies are necessary to validate and confirm this hypothesis.

There are several limitations to the current investigation. While our study provided valuable initial insights into the safety profile of Agex Fill Volume®, we did not include established in vitro methods for assessing mutagenicity and genotoxicity, such as the bacterial reverse mutation assay. Incorporating these standardized toxicological tests in future work would strengthen confidence in the safety evaluation of this low BDDE formulation. The current study was not a randomized controlled trial with a comparator arm, which means it cannot provide information on the efficacy of Agex Fill Volume® compared to other HA-based fillers for lip augmentation. The study design used an open-label approach to capture current clinical practice data, but this can introduce biases as investigators and subjects were not blinded [8,22]. Although the study was limited by the fact that assessments were performed by the treating investigators, blinding is often impractical in studies evaluating filler treatments [7]. The correlations between the low BDDE content of Agex Fill Volume® and efficacy and safety outcomes are speculative, and further studies are needed to confirm this relationship. *I*n vitro results, while conducted under controlled conditions, may not always reflect real-life situations. The enrollment of only one male participant may limit the generalizability of the findings to the male population. Finally, the initial post-procedural assessment was conducted immediately subsequent to the filler injection. Under such conditions, the observed effects may be attributable not solely to the filler but also to post-procedural edema. However, it is a common observation that patients frequently evaluate their appearance in the immediate aftermath of lip augmentation. Given that the HMS constituted a vital endpoint in our study, appraising PROs immediately following the procedure presented a distinctive opportunity to gauge the instantaneous emotional response. To ensure a comprehensive analysis, we additionally gathered data regarding the objective efficacy of the treatment at this time point.

In summary, our findings demonstrate that Agex Fill Volume®, a HA-based filler with low BDDE content, may offer a safe and effective solution for lip augmentation in real-world aesthetic practice. Our findings provide critical information for aesthetics practitioners, facilitating the making of knowledgeable choices and setting attainable expectations regarding the efficacy and safety of HA-based fillers with low BDDE content.

## Conclusions

Our findings demonstrate that Agex Fill Volume®, a HA-based filler with low BDDE content, may offer a safe and effective solution for lip augmentation in real-world aesthetic practice. Our current data provide critical information for aesthetic practitioners, facilitating the making of knowledgeable choices and setting attainable expectations regarding the efficacy and safety of HA-based fillers with low BDDE content.
